# Properties of Geopolymer Mixtures Incorporating Recycled Ceramic Fines

**DOI:** 10.3390/ma17081740

**Published:** 2024-04-10

**Authors:** Katarzyna Kalinowska-Wichrowska, Edyta Pawluczuk, Filip Chyliński, Hwa Kian Chai, Magdalena Joka Yildiz, Aleksandra Chuczun, Stanisław Łuniewski

**Affiliations:** 1 Faculty of Civil Engineering and Environmental Sciences, Bialystok University of Technology, 15-351 Bialystok, Poland; m.joka@pb.edu.pl (M.J.Y.); aleksandra.chuczun.107614@student.pb.edu.pl (A.C.); 2 Building Research Institute, Filtrowa 1, 00-611 Warsaw, Poland; f.chylinski@itb.pl; 3 School of Engineering, University of Edinburgh, Edinburgh EH9 3FG, UK; hwakian.chai@ed.ac.uk; 4 Faculty of Economic Sciences, Eastern European University of Applied Sciences, Ciepła 40, 15-472 Bialystok, Poland; astwa@astwa.pl

**Keywords:** recycled binder, ceramic fines, geopolymers, activator, heat curing

## Abstract

This research aimed to optimize the production conditions for geopolymer matrices by investigating the combination of heat curing conditions and the incorporation of recycled ceramic fines (CFs) as a partial replacement material for fly ash (FA). The obtained physical and mechanical properties of the composites confirmed the positive impact resulting from increasing the curing temperature from 65 °C to 85 °C and using CFs in the amount of 37.5% as a replacement for FA. The results were from laboratory tests performed to evaluate compressive strength, bending strength, bulk density, and water absorption of the geopolymer mixes. In addition, microscopic observations and porosity assessment were also performed, which confirmed that a further increase in the replacement of FA by CFs causes an increase in the porosity of the mixes and, thus, a decrease in all the assessed properties that are relevant to their practical use.

## 1. Introduction

In line with the European Green Deal strategy adopted in 2019, the European Union has established ambitious targets aimed at slashing greenhouse gas emissions by 55% by the year 2030 and attaining carbon neutrality by 2050 [1]. To fulfill these objectives, the construction industry must explore alternative materials that offer greater sustainability. Concrete, which is the most widely used construction material globally, second only to water, places immense demands on natural resources and contributes significantly to greenhouse gas emissions [2,3]. Notably, the production of clinker, a critical component of concrete, entails substantial carbon dioxide emissions. Approximately 830 kg of CO_2_ is released into the atmosphere for each ton of clinker produced, constituting around 5–8% of total industrial CO_2_ emissions [4]. The cement industry bears a considerable responsibility for CO_2_ emissions, thereby being a prominent contributor to climate change [5]. Indeed, CO_2_ emissions alone contribute to about 65% of the global warming challenge among all greenhouse gases [6]. Efforts to optimize the clinker production process have been ongoing to mitigate the carbon footprint; however, there are inherent limitations to how much further these emissions can be reduced [7].

An alternative for cementitious composites is geopolymers comprising alkali-activated fly ash, which has been considered a substitute for ordinary Portland cement (OPC). Geopolymers were first described by Davidovits [8] as inorganic materials rich in silicon and aluminum becoming cementitious by reacting with alkaline activators, which are usually a combination of a hydroxyl sodium hydroxide (NaOH) or potassium hydroxide (KOH)) and a glassy silicate (sodium silicate or potassium silicate). To achieve comparable strength to ordinary Portland cement (OPC) concrete, it is obligatory to cure geopolymers at an elevated temperature of between 40 and 80 °C for at least 6 h [9,10]. The range of reported CO_2_-e values for geopolymer concrete compared with OPC is estimated to be 80% less than OPC [11]. McLellan et al. 2011 [12] showed that geopolymers have great potential to reduce the impact of cement production on climate change. They estimated that the proposed ‘typical’ Australian geopolymer product would have 44–64% lower greenhouse gas emissions than OPC but that the cost of producing these geopolymers could be up to twice as high as OPC. They also indicated that these benefits could only be achieved by using the most appropriate source of raw material and the lowest transportation costs. The wide range of potential variables leads to a very large range of results when comparing the emissions of both concretes. Emissions from geopolymer concrete can be 97% lower to 14% higher compared with OPC. Therefore, each application of geopolymers must be assessed in terms of its specific location and source of raw materials. Turner and Collins 2013 [13], based on their analyses, also proved that differences in CO_2_ emissions result from whether the extraction, processing, and transport of raw materials are included in the calculations. Their estimates take into account the significant amount of energy used in the production of alkaline activators. They compared the CO_2_-e footprint generated by concretes containing geopolymer binders and 100% OPC concrete. They obtained results that showed that the CO_2_ footprint of geopolymer concrete was approximately 9% lower than that of OPC concrete. This is much less than expected.

Due to the decreasing availability of good-quality fly ash and blast furnace slag, opportunities are being sought to use other waste-containing pozzolanic oxides, which can be used as their replacement in geopolymers. Toniolo et al. 2018 [14] used waste glass as a silica supplier to avoid using water glass solution as a chemical activator. It has been proven that soda-lime glass cullet can be a substitute for commercially available sodium silicate solutions commonly used for the production of geopolymers. Additionally, the mechanical properties of the obtained geopolymers increased with the increase in the molar concentration of the activating solution. Recycling and re-using CDWs as geopolymer sources can offer a sustainable solution for reducing their ecological impact and decreasing OPC demand. More recent studies showed an excellent opportunity for CDWs to be used as aluminosilicate source materials for geopolymer binders [15]. The production of construction and demolition wastes (CDWs), particularly from ceramic and brick materials, is rising globally because of the extensive reconstruction and renovation of older buildings. These activities account for about 45% of overall CDW [16,17,18]. According to statistics, global ceramic tile production exceeds 10 million square meters annually [19]. Estimates suggest that roughly 15 to 30% of this output ends up as unused waste that accumulates in landfills. Ceramics have demonstrated exceptional resistance to biological degradation and can serve as substitutes for certain pozzolans. Their inclusion enhances concrete’s mechanical properties and durability owing to their silicoaluminate content [20,21]. Geopolymer concretes (GPCs) modified with ceramic waste additives have a denser structure with fewer pores and microcracks. The developed compositions apply to the restoration of facades of buildings and structures [22]. Aly et al. [23] examined the potential of using ceramic waste powder (CWP) in GPC using mortar specimens. The effects of air and water curing at 60 °C were investigated. Mixing with 40% slag and 60% CWP achieved 40 MPa strength after seven days. A high percentage of superplasticizers (4%) was used for flowability. The authors reported that CWP has excellent potential for use in GPC [24]. The ceramic waste studied in [16] was produced during the polishing of tile products, meaning that there was no additional energy being consumed relative to the additional process needed to crush waste ceramic tiles to derive powder forms. The main objectives were two-fold: to develop an economical, medium-strength, and feasible M35-class GPC by identifying the optimal CWP replacement for fly ash (FA) as a binder. For this purpose, two types of CWP were investigated. A 100% FA-based GPC was prepared as the base mixture, and 10, 15, and 20% of the FA were replaced with CWP. To minimize GPC cost, lower alkaline liquid-to-binder ratios (A/B) and ambient temperature curing was used. The experiments were performed at an ambient temperature of 35 ± 2 °C [24]. The key factors providing higher compressive strength at ambient temperature for the reference mix and the mix with 15% ceramic replacement were the large binder surface area, high ambient temperature, and the presence of CaO in the wall-tile ceramic waste WCWP. The larger surface area of the binders and prolonged curing at room temperature provided higher compressive strength of GPC with FA [24,25]. Chindaprasirt and Rattanasak (2017) reported improved late-age strength of GPC with FA cured at 35 °C for 72 h compared with curing at 65 °C for 24 h [26]. The extended curing period improved the strength of dust-based ceramic geopolymer bricks. However, high curing temperatures had a degradative effect on the strength properties [26]. The study presented by Silva et al. 2019 [27] reported on the optimization analyses carried out to determine the proper production conditions of fired clay brick (FCB) and natural pozzolana (NP)-based geopolymers. The results indicated that high compressive strengths of up to 37 MPa and 26 MPa can be obtained for FCB- and NP-based geopolymers, respectively, when the proper production conditions are employed. The optimum alkaline solution for FCB consisted of Ms = 0.60, Na_2_O content of 8%, and water/binder ratio = 0.27, with oven curing conditions of between 65 and 80 °C for 7 days. On the other hand, NP-based geopolymers with the highest mechanical properties were obtained with an alkaline solution composed of Ms = 1.08, 8% Na_2_O content, a water/binder ratio of 0.52, and curing in an oven at 65 °C for 7 days. The methodology for the optimization of production conditions of geopolymer matrices validated in that study demonstrated consistent results and, therefore, could be applied for the analysis of the production process of geopolymers based on other aluminosilicate sources [27]. Ceramic is a useful material for improving the strength and durability of concrete. This is because ceramic contains silico-aluminum crystalline materials. However, the recycling of ceramic waste in the construction industry (and in the production of geopolymers) is still in its early stages and is being performed in very small quantities [6].

Our research, as reported in this article, aimed to find the best combination of heat curing conditions and content of ceramic fines (CFs) as a part of an FA-based binder system to produce geopolymer concrete with desirable engineering properties suitable for general construction applications. Our research also contributes to the broader goal of reducing the environmental impact of the construction industry by promoting the use of waste materials as a substitute for standard pozzolanic binders.

## 2. Materials and Research Methodology

### 2.1. Characteristics of the Raw Materials

#### Ceramic Fines

**Figure 1:**
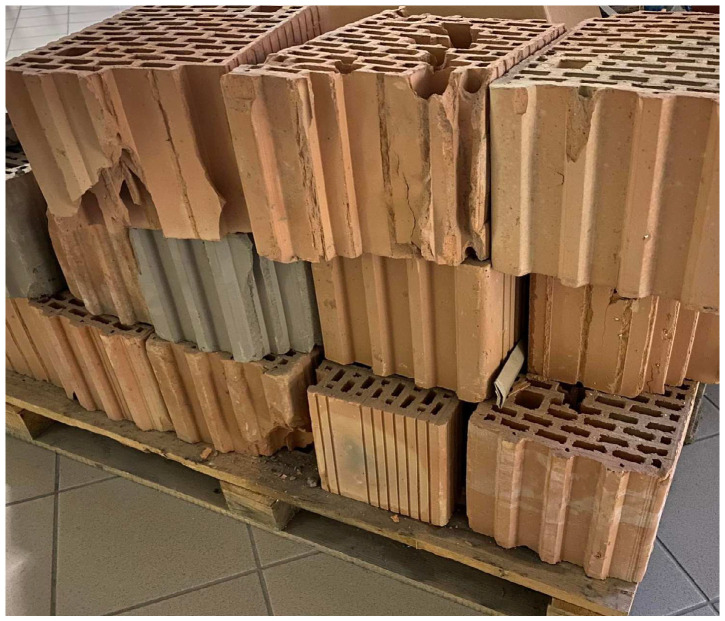
Ceramic blocks LPW 25 250/380/238 used in crushing process.

**Figure 2:**
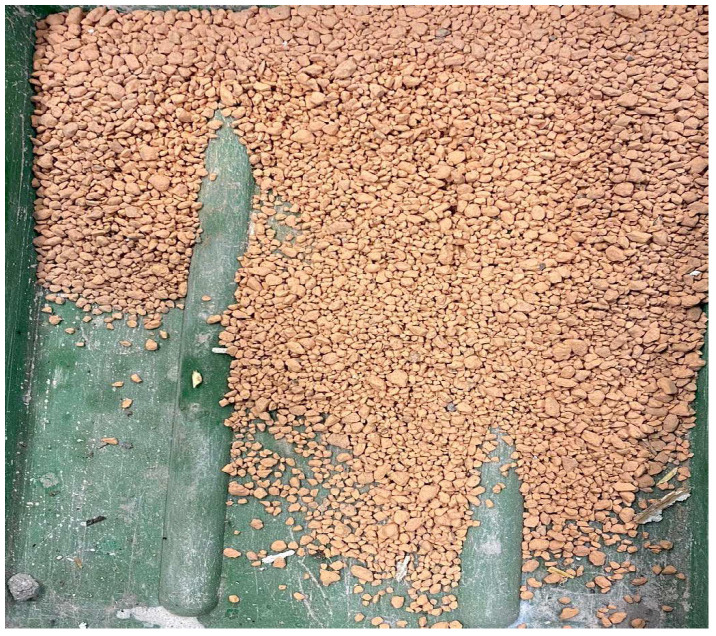
Aggregate received from crushing ceramic blocks, fraction 0–4 mm.

**Figure 3:**
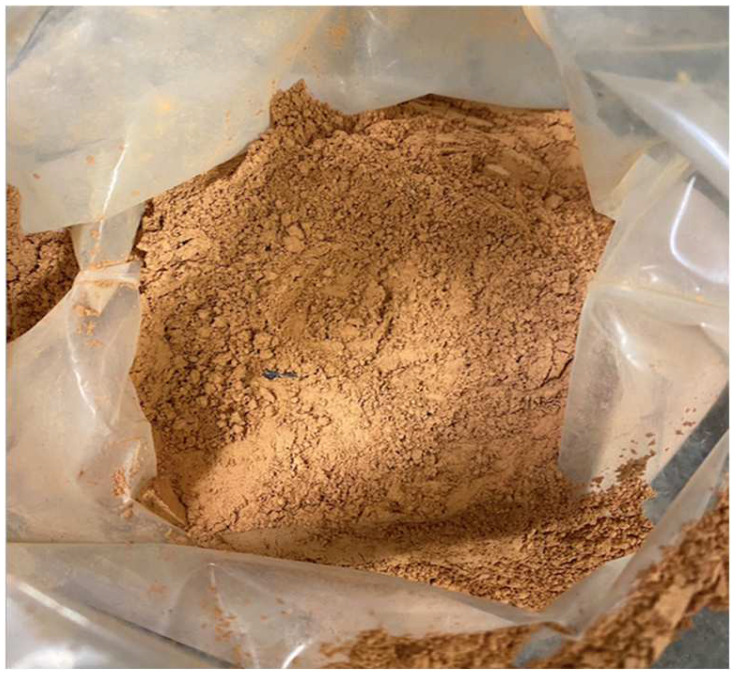
Ceramic fines after ball milling to fraction <0.063 mm.

The ceramic fines used in the research were obtained by recycling ceramic blocks, as shown in Figure 1, by crushing in a jaw crusher to grain sizes smaller than 4 cm (Figure 2). Then, the obtained material was ground in a ball mill until a fraction of 0.063 mm was obtained. Figure 2 shows ceramic aggregate with a fraction of 0–4 mm, and Figure 3 shows the ceramic fines used for testing, which were obtained from the addition of a fraction of 0–4 mm in a ball mill. The ceramic blocks (Figure 1), type LPW 25 (ceramic wall block), were delivered from the Lewkowo Company, Lewkowo, Poland. Ceramic masonry elements were used for walls, columns, and partitions. Regularly shaped, vertically drilled with a system of tongues and grooves.

The FA used met the requirements of the standard EN 450-1:2012 [28]. It is a very fine-grained powder that was obtained by burning coal dust. Fly ash used to produce a given mixture came from a heat and power plant in Poland. The chemical composition of the CFs and FA is collected in Table 1.

Fine ceramic particles consist mainly of aluminum, iron, and silica compounds and can be used as a binder in the production of geopolymer mortars instead of FA. The main properties of ceramic dust and fly ash are presented in Table 2.

**Figure 4:**
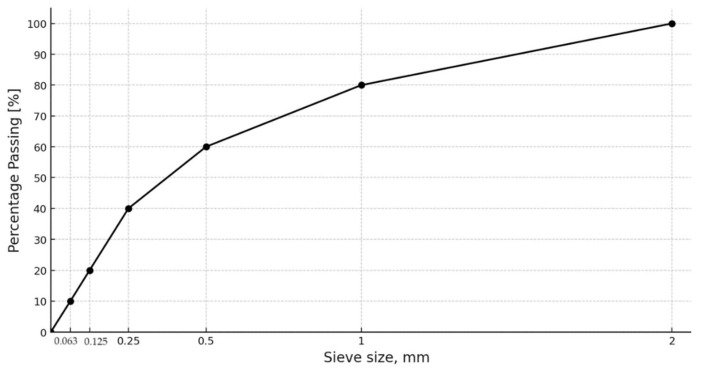
Sand sieve analysis curve.

Standard sand with a fraction of 0–2 mm was used to prepare the samples. Figure 4 shows the sieving curve of the sand.

The alkaline activator used to prepare the samples was a solution of aqueous sodium silicate and sodium hydroxide of concentration 10 M. The silicate solutions (Na_2_SiO_3_) were mixed with sodium hydroxide (NaOH) in a mass ratio of 2.5. The use of NaOH was informed by research elsewhere, which demonstrated that the inclusion of NaOH could result in greater compressive strength of geopolymer mixture compared with using KOH [29]. The alkaline activator was added to the mixture in the amount of 55% of the binder weight to maintain the workability of the fresh mortar.

### 2.2. Research Methods

Geopolymer mortar samples (40 × 40 × 160 mm prisms) were prepared following EN 196-1:2016 [30]. The flexural and compressive strength tests were performed according to EN 196-1:2016. The water absorbability test was performed by determining the percentage increase in the weight of the specimens saturated with water in relation to the weight of the specimen in the dry state. After 28 days of curing, 3 samples from each series were selected to be dried in the oven at a temperature of 85 °C. The samples were first weighed to determine their initial mass; then, they were placed in the oven to dry until constant mass was reached. After that, the samples were submerged in water until constant weight was obtained. The volume density in the dry state and the water-saturated state were determined based on EN 1015-10:1999 [31].

### 2.3. Pore Distribution—Mercury Intrusion Porosimetry (MIP)

Porosimetry test was conducted for the samples, aiming at examining how the porosity of geopolymers would change depending on the curing temperature and also the content of recycled CFs. Pore size distribution test was also carried out to discover the changes in pore sizes caused by varying curing temperatures and CF content.

The pore size distribution was analyzed using Quantachrome Poremaster, (Anton Pear Group, Boynton Beach, FL, USA) which allowed for measuring open porosity in the range of 3.6 nm and 500 μm. For MIP analysis, cores with a diameter of 25 mm were drilled from mortar prismatic samples and cut to obtain a height of 35 mm. Such cores were dried at 40 °C for 24 h before being soaked in 99% ethanol and vacuumed. After removing the ethanol, the samples were dried for 24 h at 40 °C. From each of the mixtures studied, three samples for MIP test were prepared.

### 2.4. Scanning Electron Microscopy (SEM)

Microstructural analysis was conducted on the GPC samples to examine the effect of varying curing temperature for a constant CF content. This analysis aimed to elucidate the reasons behind the observed enhancement in mechanical properties, concurrent with a decrease in volume density and alterations in total porosity. Samples containing 37.5% of CFs and cured in various temperatures were investigated (samples: 7, 8, 9).

The microstructure of mortars was analyzed using the SEM model Sigma 500 VP produced by ZEISS (Carl Zeiss Microscopy GmbH, Köln, Germany). BSE (backscattered electron) images were collected. Microanalysis was performed using EDX (energy-dispersive X-ray spectroscopy) detector model Ulitim Max 40 produced by Oxford (Oxford Instruments, High Wycombe, UK). For SEM analysis, thin slices were cut from the middle of each type of mortar bar perpendicular to the trowelling surface. The slices were trimmed to achieve surfaces measuring 20 × 20 mm in dimensions. Samples were dried and put into resin under vacuum. The next step was grinding and polishing samples to receive the proper surface for SEM-EDX examinations. The procedure of sample preparation is described widely in previous publications [32]. Before SEM examinations, the samples were gold evaporated.

## 3. Design of the Experiment

### Selection of Variables and Development of the Experimental Plan—With Ceramic Fines

The research was based on a full 2-factor design with 3 levels of variability. To carry out experimental tests, a total of 12 series were made with different contents of ceramic fines and different hardening temperatures. The ranges in variability are shown in Table 3.

Table 4 shows the distribution of the variable amount by the series number. A total of 12 series were prepared.

Upon the basis of the above-mentioned variables, an experimental plan consisting of 12 sample series was established. Table 5 shows the composition of the geopolymer mortars depending on the percentage of ceramic fines. The initial amounts of components were assumed based on the composition of standard cement mortars. The composition of mixes was designed with a constant amount of standard sand, alkaline activator, and activator/(fly ash + ceramic fines) ratio. FA was replaced with CFs in amounts from 0% to 50% by weight.

The preparation of the mortar sample began with the weighing of individual components on a laboratory balance with an accuracy of 0.01 g. Initially, the dry ingredients—FA, CF particles, and standard sand—were added to the mixer. They were mixed at a low speed for ca. two minutes. Subsequently, the activator was gradually added. The mixing was continued for three minutes. Samples were formed with the dimensions of 40 × 40 × 160 mm.

**Figure 5:**
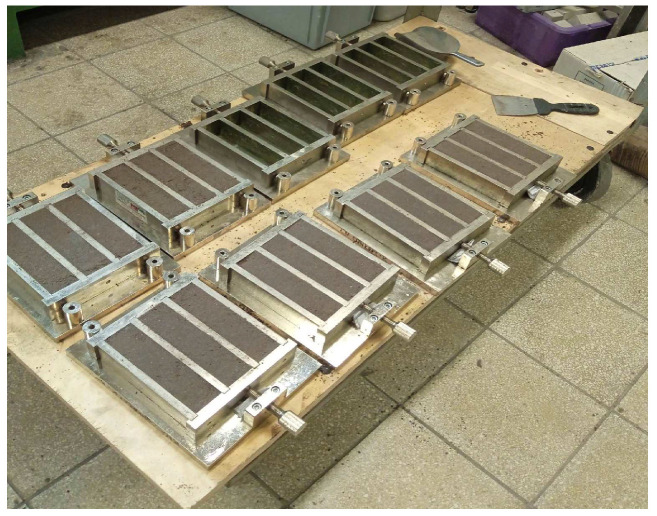
Preparation of geopolymer mortar samples.

The samples, which were formed in three-part molds, are represented in Figure 5.

After forming the samples, they were placed in a laboratory oven for 24 h at 65, 75, or 85 °C, depending on the research series. The samples were heated to accelerate the mortar-hardening process. After demolding, the samples were cured in air-dried conditions. After 28 days of curing, tests were conducted to evaluate physical and mechanical properties.

## 4. Test Results and Analysis

### 4.1. Flexural Strength of Geopolymer Mortars after 28 Days

**Figure 6:**
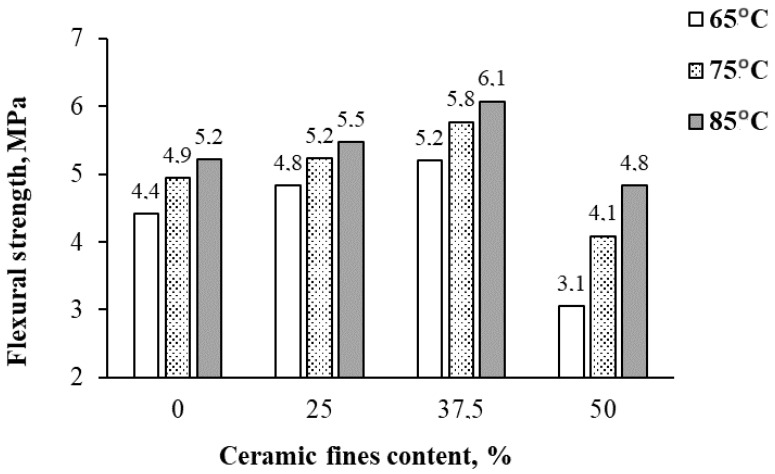
The average test results of flexural strength after 28 days.

**Figure 7:**
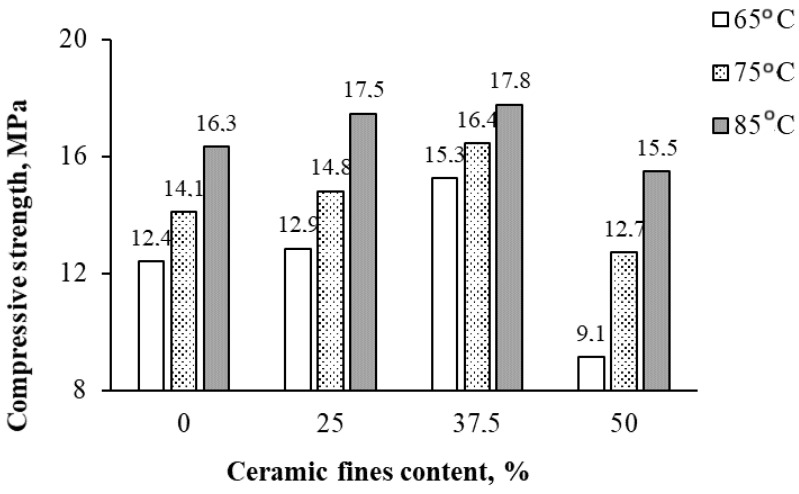
The average test results of compressive strength after 28 days.

**Figure 8:**
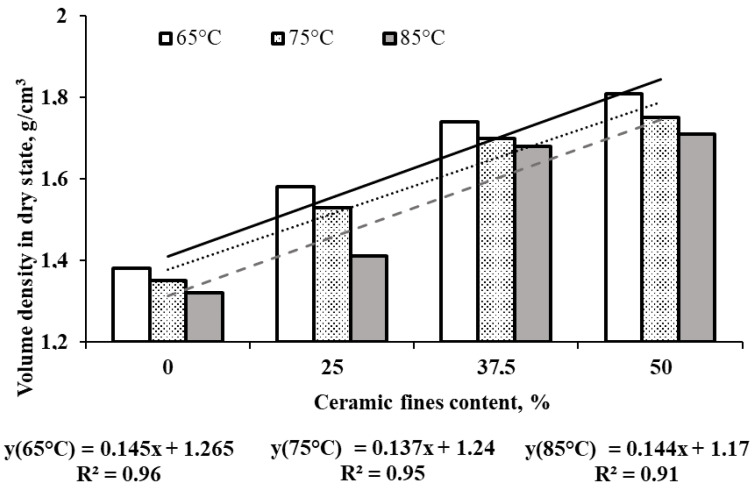
The average test results of volume density in a dry state.

**Figure 9:**
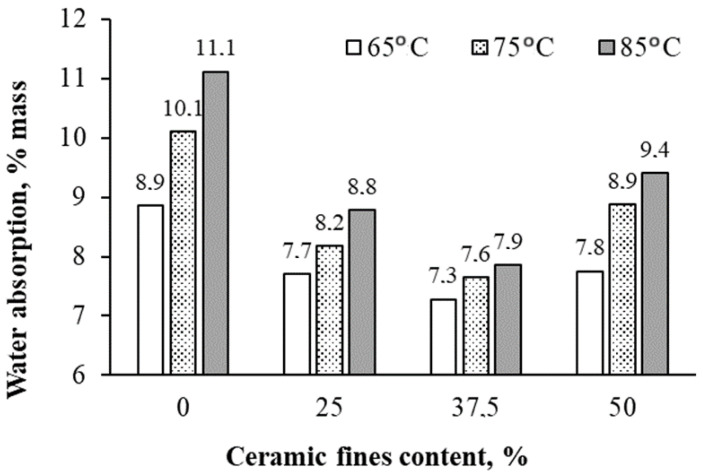
The average test results of water absorption after 28 days.

In Figure 6, Figure 7, Figure 8 and Figure 9, the average results of the geopolymer mortar properties tests for the individual series of the experiment were shown. Compressive strength, flexural strength, volume density in a dry state, and water absorption after 28 days of curing were determined.

The average results of the flexural strength tests for each series after 28 days of curing are shown in Figure 6.

As depicted in the flexural strength results of the composites (Figure 6), elevating the heating temperature from 65 °C to 85 °C led to an average flexural strength increase of approximately 16%. Moreover, augmenting the CF content from 0% to 25% and 37.5% yielded average flexural strength increases of 9% and 18%, respectively. However, escalating the CF content to 50% of the FA mass resulted in a decline in flexural strength by up to 30% compared with the control series 1CF0_65.

A similar advantageous effect of incorporating CFs into the geopolymer mortar was observed in terms of compressive strength (Figure 7). Increasing the CF content from 0% to 25% and 37.5% of the FY mass resulted in a respective 4% and 23% increase in compressive strength compared with 1CF0_65. Fe_2_O_3_ and MgO in CFs could increase the geopolymer specimen’s unit weight, compressive strength, and flexural strength, which was also reported by [33]. However, incorporating 50% CFs into the FA mass led to a decrease in compressive strength despite raising the heating temperature of the composites from 65 °C to 85 °C by an average of approximately 14% compared with the CF-free series. Decreasing compressive strength by using 50% to 70% ceramic waste was also shown by Huseien et al. [21]. The authors [21] explained this by an increase in the share of silica and a decrease in the share of calcium in the mixture, which led to the formation of a smaller amount of C-A-S-H gel.

With the CF content rising from 0 to 50%, the bulk density increased by about 30% compared with the control series 1CF0_65. Fine recycled ceramics, abundant in Fe_2_O_3_ and SiO_2_, can stimulate the formation of a new CSH gel in the geopolymer mortar, as evidenced by the notable changes in density (Figure 8) [34]. Elevating the heating temperature to 85 °C resulted in an approximate 6% decrease in bulk density. The addition of CFs contributed to structure sealing and composite density enhancement. However, it is worth noting that raising the heating temperature to 85 °C generally led to increased porosity and decreased density of geopolymers, as supported by the water absorption results (Figure 9). Water absorption of geopolymers decreased with increasing CF content from 0 to 37.5% (on average by approximately 2 pp compared with the CF_0 series) and only slightly increased with a 50% CF content in the FA mass. Heat treatment provides faster dissolution of silica and alumina species and also accelerates the degree and rate of polycondensation, which leads to the production of stronger gels at an early age of the composites and makes mixtures stronger and denser. Elevated curing temperatures can effectively improve the mechanical properties of geopolymers but within a limited range. Exposure to very high temperatures or extended curing periods may adversely affect the properties of geopolymers. This is mainly due to the loss of moisture needed for polymerization, as some of the water needed for the polymerization process easily evaporates, preventing the formation of final gels and leading to the formation of cracks and shrinkage during drying [15].

### 4.2. Porosimetry Test Results

**Figure 10:**
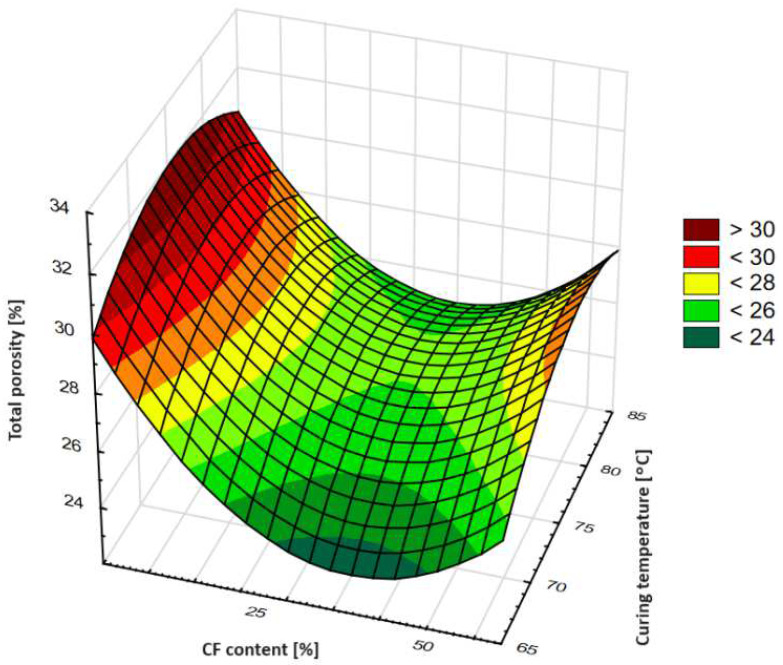
Total porosity of tested geopolymers.

Figure 10 presents a plot of the total porosity of tested geopolymers as a function of curing temperature and the content of ceramic fines.

Analyzing the results of the total porosimetry tests of the geopolymer samples, a minimum function can be observed from the 3D plot shown in Figure 10. This extremum relates with the geopolymer mixtures with the lowest total porosity. The geopolymer mixture with the lowest total porosity contained about 35% CFs. The shape of the curve with constant content of ceramic fines at 35% also had an extremum, which shows that the temperature of curing does not have a linear regression but is more parabolic, with a maximum at about 75 °C, and the slopes of the curve lessening at 65 and 85 °C. The concentration of CFs affects the total porosity of the composite more significantly than curing at temperatures in the range of 65–85 °C studied.

Through analyzing the influence of CF inclusion and FA replacement at a constant temperature of curing, it was found that increasing the amount of inclusion caused a decrease in total porosity at an amount of about 35%, but further increasing its amount caused an increase in total porosity.

**Figure 11:**
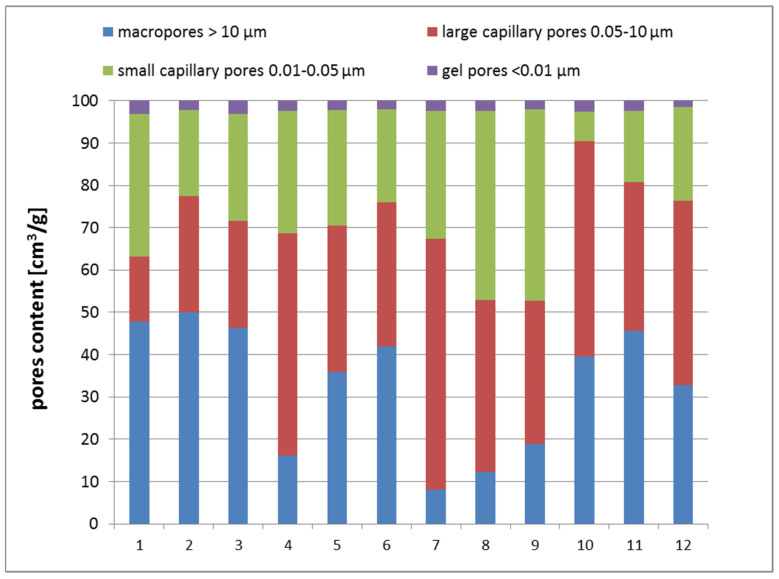
Content of various types of pores in geopolymers in each of the 12 sample series.

Figure 11 presents the content of various types of pores in the geopolymers. Examining the results of samples 7, 8, and 9, which had the same amount of CF replacement level (37.5%) but were cured at different temperatures (65, 75, and 85 °C respectively) and had the lowest total porosities, it was found that increasing the temperature of curing increased the number of large macropores (above 10 µm) and also small capillary pores (0.01–0.05 μm), although the amount of the large capillary pores (0.05–10 μm) decreased and the amount of gel pores (under 0.01 μm) remained almost constant.

By examining the variations in pore size distribution concerning the ceramic filler content, using samples cured at a consistent temperature of 75 °C (samples 2, 5, 8, and 11), it was revealed that the inclusion of CFs initially reduced the number of macropores. Simultaneously, it increased the presence of small and large capillary pores while the quantity of gel pores remained relatively constant. It was also found that a further increase in the CF replacement level beyond 37.5% caused a rapid increase in the number of macropores and a decrease in small capillary pores. According to Aredes et al. 2015 [35], geopolymers treated at 60 °C for 24 h show the smallest pores and the best compressive strength. Pores may be created by air bubbles introduced during mechanical mixing or may be trapped inside the geopolymer during formation or in the space left by evaporated water molecules. A temperature of ~80 °C is useful because all materials are easier to dissolve, but the samples have lower compressive strength due to increased porosity, which is noticeable in this case for series 10–12. There was an increase in the number of large pores and an increase in water absorption compared with the series containing 25 and 37.5% CFs and, at the same time, a decrease in compressive strength. On the other hand, the samples treated at 80 °C had lower compressive strength due to increased porosity. Heat treatment ensures faster dissolution of silica and alumina species and also accelerates the degree and rate of polycondensation, leading to the formation of stronger gels at an early age, thus making mixtures stronger and denser. Although elevated curing temperatures can effectively improve the mechanical properties of geopolymers to a certain extent, exposure to very high temperatures, as in this case, curing at 80 degrees for 24 h, adversely affected the properties of geopolymers and caused a decrease in mechanical properties. This was mainly due to the loss of moisture needed for polymerization, as some of the water needed for the polymerization process can easily evaporate, preventing the formation of final gels and leading to the formation of cracks and shrinkage during drying [15]. In the series containing 50% ceramic fines, probably due to the high content of this waste and the increase in the hardening temperature, not all components of the precursor reacted in the polycondensation process. This may be evidenced by a slight change in pore content compared to series 7, 8, and 9 but much lower compressive strength and increased bulk density.

### 4.3. Microstructure

**Figure 12:**
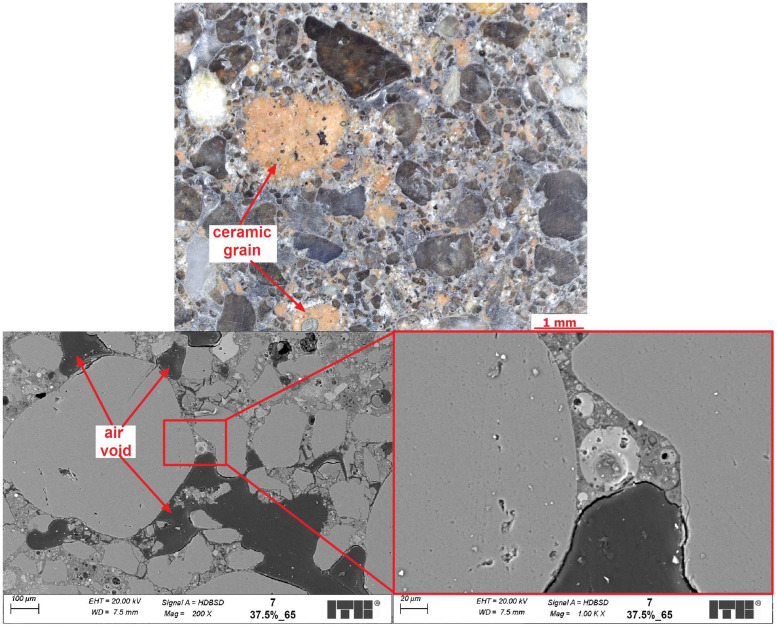
Sample 7 (temperature of curing −65 °C; the replacement level of CFs is 37.5%).

**Figure 13:**
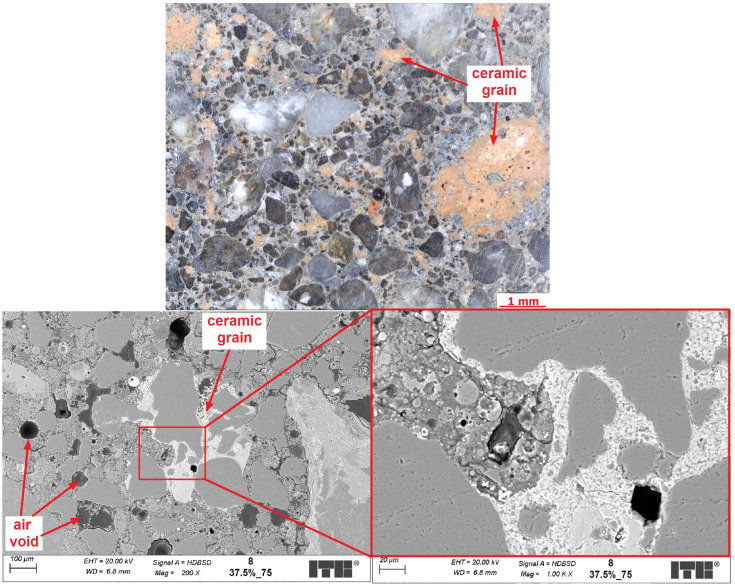
Sample 8 (temperature of curing −75 °C; the replacement level of CFs is 37.5%).

**Figure 14:**
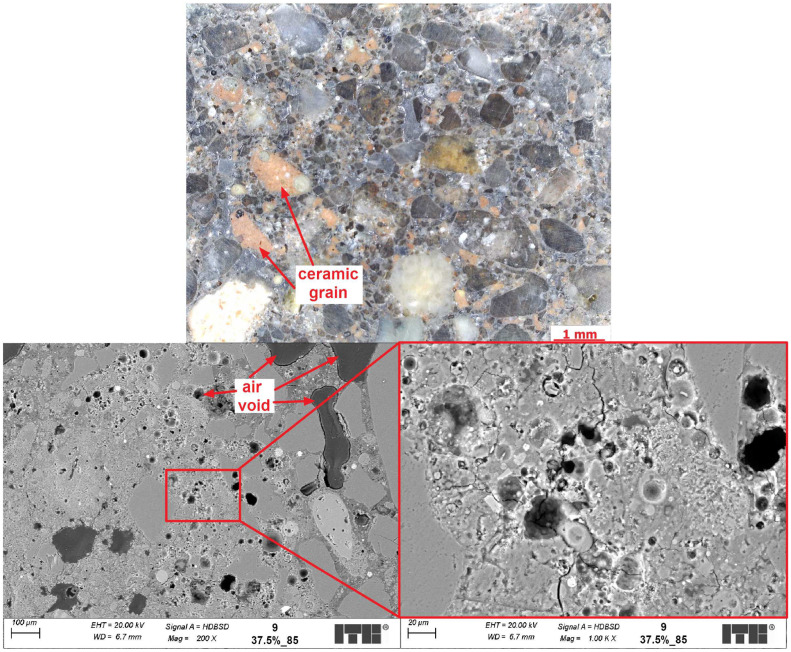
Sample 9 (temperature of curing −85 °C; the amount of ceramic fines 37.5%).

The objective of the microstructural analysis was to compare the differences in the microstructure of composites with identical compositions but cured at varying temperatures. SEM examinations might also help to discover the causes of the observed increase in mechanical properties with simultaneous decrease of volume density and might help to explain the cause of minimum total porosity of geopolymers cured at 75 °C compared with other temperatures. Figure 12, Figure 13 and Figure 14 present examples of the microstructure of geopolymers containing 37.5% CFs and cured at various temperatures.

It can be seen from the microscopic images that the samples have quite porous microstructures but with well-distributed grains of FA and also CFs as part of the matrix. Larger particles of fly ash did not fully react to form the C-A-S-H phase. It was mainly caused by the seal layer of products, which stopped any further reaction. It was a similar effect to Portland cement composites, where clinker relicts occurred in the cement matrix. The C-A-S-H phase in all examined composites was well developed, but its porosity seemed to differ from each other depending on the temperature of curing. The rising temperature of curing from 65 to 75 °C caused a sealing effect on the microstructure, which was visible from the SEM images, especially in the amount of macropores. In the observed areas of the samples, the changes were significant. However, none of the SEM semi-quantitative measurements were carried out. So, this might be subjective. Stronger arguments come from the results of the porosity test. However, increasing the temperature of curing further to 85 °C seemed to have caused the formation of some new, spherically shaped macropores in the microstructures. The formation of new macropores might be caused by the transformation of the C-A-S-H phase into its denser form, which resulted in a decrease in its volume and caused the formation of air voids. This hypothesis, however, requires further investigation. In addition, the analysis results also indicate differences in the transition zone between CFs and the geopolymer matrix when the samples were cured at different temperatures. In the sample cured at a lower temperature, the transition zone was often porous and discontinuous, but in the sample cured at a higher temperature, the transition was more sealed up and had better contact with the ceramic grain. More sealed-up transition zones in higher temperatures and different microstructures of the C-A-S-H gel might be the cause of the better mechanical properties obtained by those geopolymers.

## 5. Conclusions

This study examined the physical, mechanical, and microstructural properties of geopolymer composites utilizing CFs as a binder replacement material for FA, subjected to different curing temperatures. The following conclusions were drawn from the series of experimental investigations:

1. CFs can serve as an effective additive for geopolymer mixtures by partially replacing FA, provided the appropriate replacement level and curing temperature are implemented.
2. Enhancement in the bending and compressive strength of geopolymer mixtures was noted, with an increase in heating temperature from 65 °C to 85 °C using 25% CFs and 37.5% FA. However, employing 50% CFs alongside elevated heating temperatures resulted in diminished strength properties.
3. The incorporation of CFs generally led to reduced water absorption in geopolymers, attributed to additional sealing of their porous structure, consequently increasing bulk density.
4. The total porosity of the tested geopolymer mixtures as a function of ceramic fines content and temperature of curing has a local extremum–minimum, at a CF content of about 35%. Curing the geopolymer samples at 75 °C seems to have the strongest effect in lowering the total porosity of geopolymers. Observed changes in the total porosity are mostly caused by the amount of macropores and capillary pores, but the amount of gel pores seems to be almost constant.
5. Increasing the temperature of curing would cause the sealing up of geopolymer microstructures initially, but a further increase in the temperature causes the formation of new macropores, which might be due to the formation of a denser C-A-S-H phase. However, this hypothesis needs further investigation to confirm.

The test results obtained from this study have opened up the possibility for extended research programs involving the use of industry-recycled CFs as part of the pozzolana materials suitable for developing sustainable geopolymer mixtures for mortar and concrete applications.

## Figures and Tables

**Table 1:** Chemical composition of CFs and FA.

Composition Percentage, %	CaO	Fe_2_O_3_	SiO_2_	Al_2_O_3_	MgO	SO_3_	Na_2_O	K_2_O	LOI
CFs	10.22	13.69	38.56	18.94	4.06	0.15	1.58	6.29	-
FA	2.14	4.97	54.60	25.30	1.80	0.37	0.84	2.80	4.37

**Table 2:** The main properties of CFs and FA.

Properties	Skeletal Density, g/cm^3^	Bulk Density, g/cm^3^	Bulk Density in a Loose State, g/cm^3^
CFs	2.82	0.76	0.71
FA	2.35	0.85	0.79

**Table 3:** Variable levels in the experiment plan.

*X*_1_	Amount of CFs	0	25	37.5	50	% of FA
*X*_2_	Temperature of curing	-	65	75	85	°C

**Table 4:** Plan of experiment.

Series	Variables	
	*X*_1_, %	*X*_2_, °C
1	0	65
2	0	75
3	0	85
4	25	65
5	25	75
6	25	85
7	37.5	65
8	37.5	75
9	37.5	85
10	50	65
11	50	75
12	50	85

**Table 5:** The composition of the geopolymer mortars depends on the content of CFs (on three samples, 40 × 40 × 160 mm).

Series	Percentage of CF Content, %	CF, g	FA, g	Activator 10 M, g	Sand, g	Curing Temperature, °C
1CF0_65	0	0	450	250	1350	65
2CF0_75	0	0	450	250	1350	75
3CF0_85	0	0	450	250	1350	85
4CF25_65	25	112.5	337.5	250	1350	65
5CF25_75	25	112.5	337.5	250	1350	75
6CF25_85	25	112.5	337.5	250	1350	85
7CF37.5_65	37.5	168.75	281.25	250	1350	65
8CF37.5_75	37.5	168.75	281.25	250	1350	75
9CF37.5_85	37.5	168.75	281.25	250	1350	85
10CF50_65	50	225	225	250	1350	65
11CF50_75	50	225	225	250	1350	75
12CF50_85	50	225	225	250	1350	85

## Data Availability

Data are contained within the article.
